# The Association Between Distance Learning, Stress Level, and Perceived Quality of Education in Medical Students After Transitioning to a Fully Online Platform

**DOI:** 10.7759/cureus.24071

**Published:** 2022-04-12

**Authors:** Rida Altaf, Michael Kling, Arielle Hough, Jibran Baig, Andrea Ball, Jessica Goldstein, Jamie Brunworth, Cassidy Chau, Marissa Dybas, Robin J Jacobs, Joshua Costin

**Affiliations:** 1 Medical School, Dr. Kiran C. Patel College of Osteopathic Medicine, Nova Southeastern University, Fort Lauderdale, USA; 2 Medical and Behavioral Research, Health Informatics, Medical Education, Dr. Kiran C. Patel College of Osteopathic Medicine, Nova Southeastern University, Fort Lauderdale, USA; 3 Department of Microbiology, Dr. Kiran C. Patel College of Osteopathic Medicine, Nova Southeastern University, Fort Lauderdale, USA; 4 Department of Medical Education, Dr. Kiran C. Patel College of Osteopathic Medicine, Nova Southeastern University, Fort Lauderdale, USA

**Keywords:** distance learning, personality type, burnout, stress, osteopathic medical students, medical education, online teaching

## Abstract

Background: Before COVID-19, preclinical medical students traditionally attended didactic lectures in in-person settings. Due to social distancing, students were required to switch to online meeting platforms, such as Zoom. For medical students accustomed to in-person interactions, these changes may add more stress to the already stressful medical school experience. Furthermore, it was unclear if students’ stress levels were related to their preference for one learning modality over another. The purpose of this study was thus to explore associations between lecture modality (synchronous Zoom lectures versus live, in-person lectures) and stress in second-year medical students after they transitioned from a face-to-face learning experience to a fully online lecture platform.

Methodology: Cross-sectional data were collected from 112 second-year medical students enrolled in a large U.S. medical school using an anonymous questionnaire delivered electronically via social media and emails. The survey contained items pertaining to students’ attitudes towards different types of lecture modalities and how they relate to personal stress. Descriptive data and Spearman’s rank correlation tests were conducted using IBM Corp. Released 2020. IBM SPSS Statistics for Windows, Version 27.0. Armonk, NY: IBM Corp.

Results: This study examined correlations between preclinical medical school lecture delivery and personality type, stress levels, attendance, and burnout. Overall, no significance was found between mode of delivery and personality type. On the other hand, the mode of delivery significantly affected stress levels, attendance, and burnout. Moderate to strong correlations were found between the item “Zoom lectures have reduced stress compared to in-person lectures” and preference for Zoom, quality of education using Zoom compared to the in-person lectures, belief that Zoom lectures should continue as part of the curriculum delivery method, staying motivated with lectures fully online with Zoom, and liking that Zoom lectures save commute time to campus.

Conclusions: Findings suggest that a fully online curriculum may play a role in reducing stress in medical students without compromising the quality of education.

## Introduction

Before the COVID-19 pandemic, preclinical medical students were traditionally recommended to attend all didactic in-person lectures. With the onset of the COVID-19 pandemic, social distancing, and masking requirements put in place, higher education learning institutions and students had to adjust to the online mode of learning, with lectures and education provided using synchronous meeting platforms, such as Zoom. It remains unclear whether the added stress of the pandemic and alterations in learning modalities, including the use of synchronous meeting platforms for lectures and engagement, would result in added stress for students attending medical school. As such, our study aimed to explore associations between lecture modality (synchronous Zoom lectures versus live, in-person lectures) and personality type, stress levels, attendance, and burnout in second-year medical students after students transitioned from a face-to-face learning experience to a fully online lecture platform.

Medical students and stress

According to the National Institutes of Health, stress is defined as the body’s response to physical, mental, or emotional tension [1]. It can cause chemical changes and imbalances in the body, creating a susceptibility to illness and disease [1]. The effects of long-term stress range widely and manifest in many different chronic disease states, including mental illnesses such as anxiety and depression [1,2], suggesting that stress for medical students has been reported to be related to a high prevalence of self-reported depression when compared to the general population. Medical students were found to experience more depressive episodes in in-person 12 months than the general population of 18 to 49-year-olds [3,4]; the test was performed on a large-scale meta-analysis which demonstrated that the prevalence of anxiety in medical students was 33.8% which can be compared to the highest 25% in the general population.

Many factors play a role in why medical students experience this stress not seen in their cohorts, including large study loads, time commitment, pressures of the clinical environment, fear of failure, and a copious number of exams [5]. The COVID-19 pandemic created new challenges for medical students, both scholastic and emotional. This major global issue forced medical schools to create an acceptable mode of medical education that had the least modification to both preclinical and clinical curricula while still ensuring students’ safety during the pandemic. The key concern associated with this change in learning dynamic was not only to ensure that the education was properly and effectively being delivered but that this format was not adding additional stress to an already stressful education process, particularly for students in their first and second years of study.

Teaching modalities

The traditional live, in-person lecture style sets the baseline standard for the quality of medical education. The lack of a traditional face-to-face learning environment may lead to distractions and difficulties focusing on lectures. The professors in Pakistan during the COVID-19 pandemic faced challenges related to the engagement of medical students with short attention spans when learning was taught on online platforms [6]. They were concerned about students marking proxy attendance while engaging in other online activities simultaneously. Live, in-person lectures guarantee that a student is receiving content, whereas online lectures may go unwatched due to a lack of self-discipline or the presence of distractions [7]. These discrepancies between modalities may lead to a decrease in the perceived quality of an online-based education style. In addition, student concerns regarding unfamiliarity with the online educational programs, technical difficulties, and possible increase in classwork due to inexperience with an online form of education may provide barriers to quality online education for students [7].

However, using both asynchronous and synchronous online teaching methods, instructors had high acceptance of online teaching and perceived that online teaching fulfilled its purpose in educating medical students during the pandemic [8]. Education through an asynchronous, recorded method allows students the flexibility to watch lectures in their own comfortable space, save time by not traveling to a site, and increase their interest and ability to focus on learning. Using this method, medical students can review lessons based on their learning needs which may enhance and promote a tailored education [8,9]. Moreover, there are reports of students who transitioned to an e-learning environment during the COVID-19 pandemic and would prefer to continue studying online or through a hybrid method (combination of face-to-face and e-learning) in the future [10].

Personality type

Personality type has been categorized by Myers and Briggs [11]. The Myers-Briggs Type Indicator (MBTI) is a test that measures one’s behavioral and personality tendencies to explain one’s perception, interaction, and decisions in the world, particularly related to introversion and extroversion [12]. Specifically categorizing individuals as introverts if they prefer to place greater attention on and receive energy from spending time in the inner world of images and ideas versus extroverts in the outer world of things and people [11]. To our knowledge, the relationship between MBTI introversion vs. extroversion as it relates to students’ preferences in lecture modality has not been thoroughly examined in medical students, aside from questionnaires to determine broader relationships between personality traits and learning styles [13,14]. 

## Materials and methods

Cross-sectional data were collected via an electronic anonymous and voluntary survey from second-year medical students enrolled in a college of osteopathic medicine in Florida, United States. Anecdotal evidence and previous research on the subject guided the creation of the survey items. The survey consisted of Likert-type items about students’ attitudes towards different types of lecture modality (synchronous Zoom lectures versus live, in-person lectures) regarding stress level, lecture modality preference, perceived quality of education with online learning, staying motivated, and attitudes toward lecture modality provided post-COVID distancing restrictions. Also included were items regarding burnout and mental health (i.e., not seeing my classmates and professors in person has negatively affected my mental health) and personality type (introvert/extrovert) based on previous Myers Briggs Type Indicator scores as it was thought that introverts and extroverts might respond differently to the learning modality transition (i.e., face-to-face to online). 

Single items related to the participant’s attitudes toward different teaching modalities (e.g., “I find it more difficult to stay motivated with this new online-style learning system such as live Zoom”; “Not seeing my classmates and professors in person has negatively affected my mental health,” and “Live Zoom lectures have reduced stress compared to live in-person lectures”) were rated on a scale of 1-4 (rarely, never, always, almost always). Single items related to the participant’s class attendance and burnout included items such as “How often have you experienced burnout since classes switched to include Live Zoom in August?” and “How frequently did you attend live, in-person lectures before the changes were implemented due to COVID-19 in August 2020?” were rated on a scale of 1-4 (not at all, rarely, sometimes, more often than not).

Sample and recruitment

Approval for this study was granted by the researchers’ university’s ethics committee for research with human subjects. The self-administered anonymous survey was administered to the entire class of 403 second-year medical students enrolled (medical school identity omitted for blind review) via an electronic link using REDCap, a user-friendly secure web application for building and managing online surveys and databases. The survey took approximately 5-7 minutes to complete. Reminder notifications were given after five days, after two weeks, and then again five days before closing data collection to promote respondent participation in and completing questionnaires and thereby reduce non-response rates.

Analysis

Of the 403 second-year students enrolled in the medical school program, 131 surveys (32.5%) were returned. Of those, 19 cases had < two-thirds (66%) of data submitted and were thus removed, resulting in a final sample size of 112 completed questionnaires (85.5% completion rate). IBM Corp. Released 2020. IBM SPSS Statistics for Windows, Version 27.0. Armonk, NY: IBM Corp. was used to analyze the data from this study after extraction from REDCap.

Sample characteristics were summarized as frequency and percentage for discrete variables. A Spearman’s rank correlation coefficient analysis was conducted to identify correlations between the variables of interest. A paired sample t-test was performed to investigate significant changes in the perceived frequency of burnout since classes switched to live Zoom sessions from in-person sessions.

## Results

Characteristics of the sample

About half (n=58; 51.8%) of the participants were women, and 48.2% (n=54) were men. Most participants (n=82; 73.2%) reported having a 15 minute or less commute to campus; 17% (n=19) reported a 16-30-minute commute, 8.8% (n=10) reported 31-45-minute commute, and < 1% (n=1) reported a 46-60 minute or more commute. Regarding attendance, 26.8% (n=30) of the participants reported they “nearly always” or “often” attended live lectures prior to the COVID-19 pandemic, whereas 2.7% (n=3) reported they “nearly always” and 0% (n=0) reported they "often" attended live lectures during the pandemic. 

Stress and attitudes toward different teaching modalities

Table 1 reports the descriptive responses of the single items related to the participant’s stress and attitudes toward different teaching modalities. Most of the participants who agreed they preferred Zoom lectures over live, in-person lectures reported that 91.6% (n=87) agreed that Zoom lectures have reduced their stress level compared to seven live, in-person lectures. The majority felt that there was no educational value lost in having Zoom lectures over live, in-person lectures and believed that Zoom lectures should continue as the lecture modality even after social distancing restrictions are lifted. 

**Table 1:** Attitudes Toward Different Teaching Modalities

Survey Item	Mean	SD	Range
I prefer Zoom over live, in-person lectures	1.76	.797	1-4
Live Zoom lectures have reduced stress compared to live in-person lectures	1.78	.744	1-4
I don’t feel that I am missing any value in my education by having live Zoom lectures rather than live, in-person lectures	1.99	.944	1-4
I believe that live Zoom lectures should continue as part of the curriculum delivery method after social distancing measures due to COVID-19 are no longer necessary	1.82	.862	1-4
I find it more difficult to stay motivated with this new online-style learning system (live Zoom)	2.64	.957	1-4
My commute affects whether I attend in-person classes or watch recorded lectures	2.53	1.115	1-4
I like that live Zoom lectures have saved me commute time to campus	1.58	.718	1-4
Not seeing my classmates and professors in person has negatively affected my mental health	2.11	.786	1-4

Attendance and burnout

Table 2 reports the descriptive results of the single items related to the participant’s class attendance pre- and post-transition to a fully online platform.

**Table 2:** Student Attendance to Live, In-person Lectures Pre- and Post-transition to Fully Online Platform

Survey Item	Almost Never (n, %)	Sometimes (n, %)	Often (n, %)	Nearly Always (n, %)
How frequently did you attend live, in-person lectures before the changes implemented due to COVID-19 in August 2020?	48 (42.9)	31 (27.7)	10 (8.9)	20 (17.9)
How frequently do you attend live, in-person lectures after the changes implemented due to COVID-19 in August 2020?	98 (87.5)	8 (7.1)	3 (2.7)	3 (2.7)

Table 3 reports the descriptive results of items asking about student burnout pre- and post-transition to a fully online platform. 

**Table 3:** Student Burnout Pre- and Post-transition to Fully Online Platform

Survey Item	Not at All (n, %)	Rarely (n, %)	Sometimes (n, %)	More Often than Not (n, %)	All the Time (n, %)
How often did you experience burnout before the format switched to include live Zoom in August?	5 (4.5%)	20 (17.9)	43 (38.4)	36 (32.1)	5 (4.5)
How often have you experienced burnout since classes switched to include live Zoom in August?	5 (4.5%)	23 (20.5)	58 (51.8)	20 (17.9)	3 (2.7)

A paired-sample t-test was conducted to compare the perceived level of burnout in participants before and after the transition from face-to-face learning to online platforms. There was a significant difference in scores for the item “How often did you experience burnout before the format switched to include live Zoom? (M= 3.15, SD=.932) and “How often have you experienced burnout since classes switched to include live Zoom?” (M=2.94, SD=.831); t (108)=2.36, p=.019. This finding indicates there was a statistically significant decrease (p<.05) in reported burnout in participants after an all-online curriculum was implemented than before (primarily face-to-face). 

Personality type

Although it was thought that introverts and extroverts might respond differently to the learning modality transition (i.e., face-to-face to online), no significant associations were found between personality type and any of the other study variables. 

Stress

Table 4 reports the significant bivariate correlations between variables. Moderate to strong correlations were found between the item “Zoom lectures have reduced stress compared to live, in-person lectures” and preference for Zoom (r=.660, p<0.01), quality of education using Zoom compared to the live-in person lectures (r=.571, p<0.01), belief that Zoom lectures should continue as part of the curriculum delivery method (r=.728, p<0.01), staying motivated with lectures fully online with Zoom (r=.466, p<.01), and liking that Zoom lectures save commute time to campus (r=.542, p<.01).

**Table 4:** Variables Correlated With “Live Zoom Lectures Have Reduced Stress Compared to In-Person Lectures” *Correlation is significant at the 0.01 level (2-tailed).

	Live Zoom lectures reduced stress
Prefer Zoom live lectures (over in-person lectures)	.660*
Believe there is no decrease in quality of education with Zoom live lectures compared to in-person, live lectures	.571*
Believe Zoom lectures should continue as part of the curriculum delivery method	.728*
Liking that Zoom lectures save commute time to campus	.466*
Easy to stay motivated with this new online-style learning system (live Zoom)	.542*

## Discussion

This study examined correlations between preclinical medical school lecture delivery and personality type, stress levels, attendance, and burnout. Overall, no significance was found between mode of delivery and personality type. On the other hand, the mode of delivery significantly affected stress levels, attendance, and burnout.

Stress and burnout

When medical students switched to online synchronous learning over zoom, stress levels were decreased as compared to in-person learning. Despite the stress of a pandemic, medical students surveyed here benefited from online learning with reduced stress. Lecture benefit was not reduced when switched to online learning over Zoom: students retained the ability to interact with the professor and other students through chat and live questions. Accordingly, the reduced stress felt by students led to decreased burnout felt by students.

Compared to other institutions, this sample of medical students from one institution may have experienced higher stress levels pre-pandemic due to a variety of factors. For example, parking challenges, campus setting, and more. In this study, students reported decreased commute time as a major stress-reducing factor. These factors should be explored further across numerous medical schools to best understand ways to mitigate student stress and burnout.

Emotional, physical, and mental exhaustion can contribute to burnout in medical students [2]. Decreased commute times and the ability to take classes and study at home (made possible by online educational platforms such as Zoom) may help decrease the physical exhaustion component of burnout. Findings from this study lead to the thought that online learning platforms can be extended as a permanent option in medical curricula, but more robust, longitudinal studies of its impact may be warranted.

Attendance

An important aspect of any educational program is student attendance. Attendance, through a variety of different methods, requires students to engage with the learning material in a synchronized fashion as the material is presented. Research has shown that the association between attendance and academic performance is not clear and is, in fact, variable [15]. Although this has led to differing opinions regarding opting for mandatory attendance in medical schools across the country, rates of attending lectures have been steadily decreasing year to year [16]. 

Findings from this study indicate that attendance and perceived academic success may have been affected by transitioning to a live zoom and video recording lecture setting. Participants reported they were more likely to attend lectures after the transition to a fully online curriculum. A possible reason for this was explored through items that were concerned with commuting; a factor mainly presents before the changes in August 2020. This study demonstrated that second-year medical students significantly appreciated the elimination of the need to commute for in-person lectures. Moreover, perceived academic success may have been due to the transition to a fully online didactic curriculum. These findings support previous research findings whereby students who had a curriculum that offered online classes and individual study options (thus decreased commute time) promoted better academic performance [17].

Some classes required mandatory attendance as they relied on a combined visual, auditory, and tactile learning experience geared towards developing skills that would not be possible without in-person instruction. Mandatory attendance policies may have influenced the perception of how the study participants rated attendance in both in-person and online synchronous Zoom lectures.

Limitations of the study

A cross-sectional, correlational survey design was used to collect data from a single institution, and generalizations cannot be made concerning changes over time. Moreover, due to the type of study, causal relationships between variables cannot be determined. As a single-site study, it is improbable that the data reflect a diverse sample of student participants. Moreover, self-report questionnaires may produce response bias, social desirability bias, and other inaccuracies. Additionally, stress due to navigating a COVID-19 world may have influenced participant responses. The study collected data electronically from the class of second-year medical students who had the first-year experience of primarily face-to-face courses and the second year of completely online learning experiences. The idea was to look at the differences in this cohort specifically, particularly regarding their stress, burnout, and perceived academic success that may have been affected by transitioning to a live-zoom and video recording lecture setting. The premise was to survey those students who had the experience of transitioning from one modality to another, as opposed to a cohort who never had the experience of face-to-face medical education. COVID-19 may have indeed acted in some way as a confounding influence. However, the conclusions drawn from this survey may be of some use in directing more focused research efforts to reveal what types of learning modalities and applied curriculum changes may help reduce stress in students, particularly those in high-stress programs such as medicine and those who are not accustomed to online learning platforms.

## Conclusions

The findings from this study suggest that students (overall) preferred online synchronous lectures via zoom compared to live, in-person didactic lectures. There has been much discussion since the onset of the COVID-19 pandemic regarding the value and efficacy of an online medical school curriculum due to the conjecture that online programs may affect engagement, increase distractions, and create technical difficulties. The format of lectures, along with other factors reviewed in other studies, highlight the advantages of online synchronous lectures. Furthermore, most of the participants agreed that Zoom lectures should continue as part of the curriculum delivery method even after social distancing regulations due to COVID-19 cease. Future research should explore the association between lecture modality, quality of education, and outcomes regarding online versus face-to-face learning environments and the impact of these modalities on student stress.
